# Fungal keratitis treated with a combination of traditional Chinese medicine and Western medicine: A case report

**DOI:** 10.1097/MD.0000000000031976

**Published:** 2022-12-02

**Authors:** Yu Huang, Juan Yu, Qinghua Peng

**Affiliations:** a Hunan University of Chinese Medicine, Changsha, Hunan, China; b The First Affiliated Hospital of Hunan University of Chinese Medicine, Changsha, Hunan, China.

**Keywords:** case report, fungal keratitis, traditional Chinese medicine

## Abstract

Currently, the primary treatments for fungal keratitis (FK) are drugs and surgery. However, drug treatment has low efficacy and many side effects, and surgical treatment is costly. Therefore, it is critical to develop a new method of FK treatment. This report describes a 56-year-old male patient with FK who was treated with a combination of traditional Chinese medicine (TCM) and Western medicine with noticeable results and few side effects.

**Patient concerns:**

The main symptoms were blurred vision in the right eye and pain. On the corneal surface, a large area of ulcer with a turbid margin was visible, along with an oral ulcer. Additionally, the patient was afraid of corneal transplantation due to financial constraints.

**Diagnoses:**

The case was diagnosed as FK. In vivo confocal microscopy is the first choice for the diagnosis of this condition. Corneal ulcer was infiltrated with numerous inflammatory cells and dendritic fungal hyphae, as determined by in vivo confocal microscopy.

**Interventions:**

Early in his illness, the patient was treated with only Western medicine, which resulted in poor outcomes and severe adverse reactions. Corneal transplantation was recommended by the first hospital. The patient was later transferred to our hospital for treatment with TCM decoction.

**Outcomes:**

After 21 days of treatment, the corneal ulcer of the patient became shallower, his vision improved, and his discomfort disappeared. Due to financial concerns, the patient and his family requested early discharge, so no follow-up disease information was obtained. However, when analyzing the disease development process in the hospital, the combination of TCM and Western medicine had obvious effects and a high level of safety.

**Lessons:**

This case report shows that TCM is safe and effective in the treatment of FK and is worthy of promotion. However, in practice, we found that TCM is better for patients with early FK, so early diagnosis of FK is crucial.

## 1. Introduction

Fungal keratitis (FK) is a kind of refractory infectious keratopathy with a high rate of blindness.^[1]^ The treatment of this disease has been the subject of extensive research and development in recent years, but its therapeutic effects are generally limited. At present, FK is treated with a combination of traditional Chinese medicine (TCM) and Western medicine. It was found that the curative effect of the combination of TCM and Western medicine for the treatment of FK is favorable, although there are few reports of its use domestically and abroad. Therefore, this case report will describe a case of FK treated with a combination of TCM and Western medicine, with the goal of providing new options for the treatment of the disease.

## 2. Case report

We present the case of a 56-year-old male patient who developed FK. This patient visited our hospital due to blurred vision and pain in his right eye for more than 20 days. He was admitted to our hospital with FK (right eye) on September 21, 2021. Before the onset of the disease, the patient had a history of corneal foreign body trauma. After the onset of the disease, he was given infusion treatment in the local hospital for 6 days (the specific treatment method is unknown), but his symptoms were not relieved. He had no history of systemic diseases, such as diabetes or hypertension.

**Figure 1.**
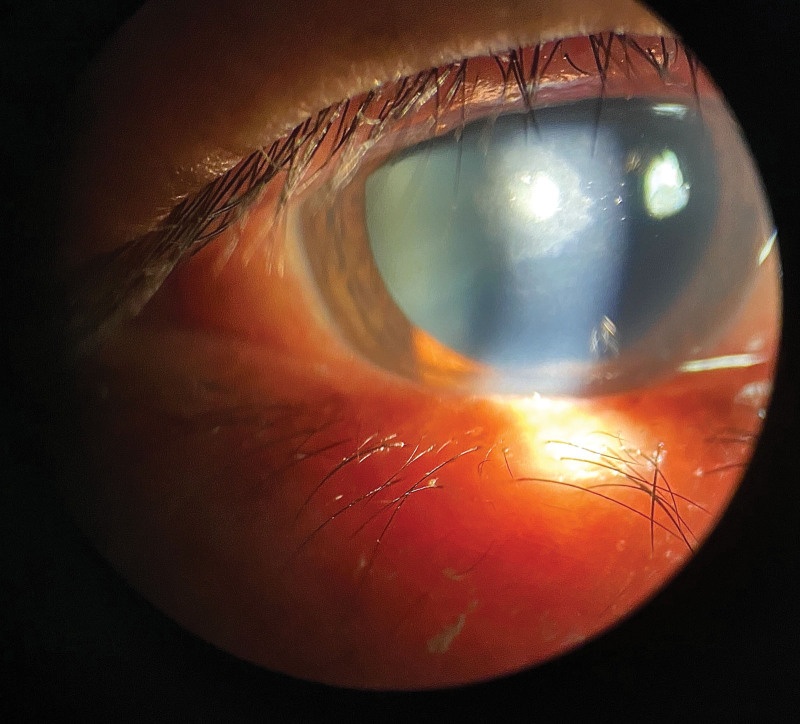
Ophthalmic examination after admission: there was a large moss-like structure in the center of the cornea that measured approximately 3 × 4 mm and reached Descemet membrane.

**Figure 2.**
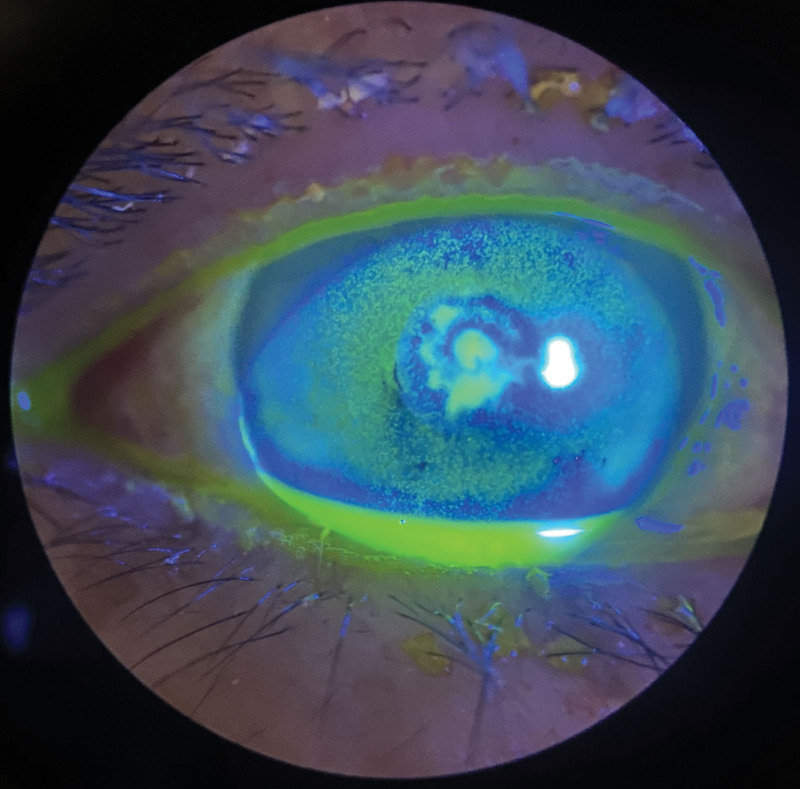
Ophthalmic examination after admission: corneal fluorescein sodium staining was positive.

**Figure 3.**
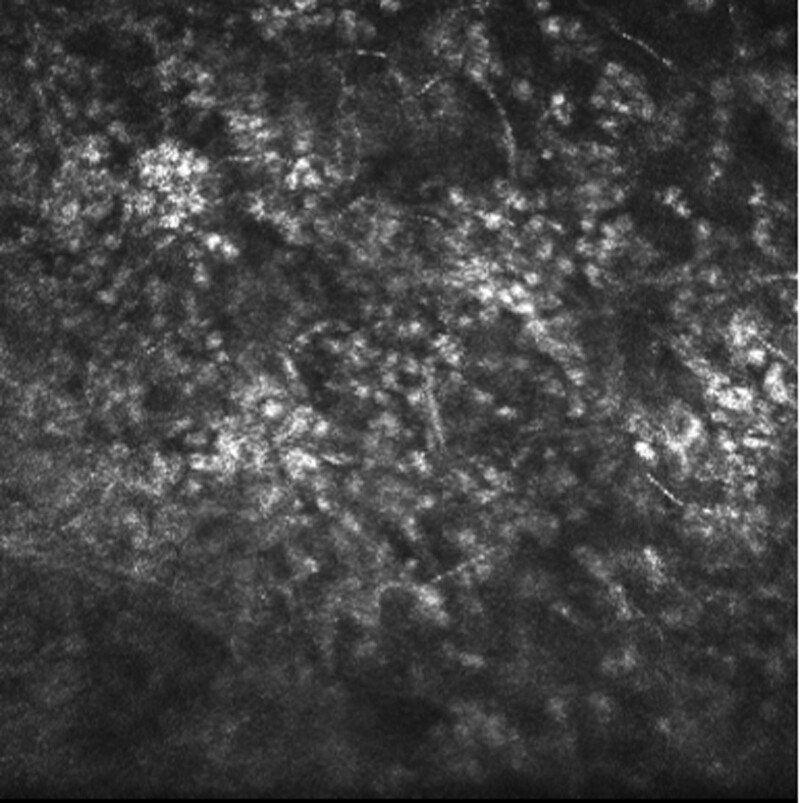
Ophthalmic examination after admission: in vivo confocal microscopy (IVCM) showed that a large number of inflammatory cells infiltrated the corneal ulcer, accompanied by dendritic fungal hyphae.

Ophthalmic examination after admission: The naked visual acuity of the right eye was 0.15, while that of the left eye was 0.5. The noncontact intraocular pressure of the right eye was Tn, while the noncontact intraocular pressure of the left eye was 10 mm Hg. The bulbar conjunctiva of the right eye was congested. There was a large moss-like structure in the center of the cornea that measured approximately 3 × 4 mm and reached Descemet membrane (Fig. 1). Corneal fluorescein sodium staining was positive (Fig. 2). The border of the corneal defect area was circular gray white opacity, while the peripheral corneal area was transparent. The anterior chamber was spacious and free of pus. The pupil was round and 4 mm in diameter. The iris texture was clear. The ocular fundus of the right eye was invisible. No obvious abnormalities were found in the left eye. In vivo confocal microscopy (IVCM) showed that a large number of inflammatory cells infiltrated the corneal ulcer, accompanied by dendritic fungal hyphae (Fig. 3). In general examination, no obvious abnormalities were found in the liver, heart, spleen, or kidney.

After admission, natamycin eye drops were administered locally every 1 hour, and pranoprofen eye drops were administered locally 4 times a day. A fluconazole injection was given intravenously. In addition, amphotericin B was injected subconjunctivally every 3 days. Based on the tongue manifestation, pulse condition, and general condition of the patient, a dampness-heat pattern was identified as the TCM syndrome type. The patient was given Sanren decoction (Xingren 15 g, Doukouren 10 g, Yiyiren 10 g, Houpu 6 g, Tongcao 10 g, Banxia 10 g, Feihuashi 10 g, Zhuye 10 g). The Chinese herbal decoction was added to Huangqin 6 g, Jinyinhua 10 g, and Lianqiao 6 g to clear heat and purge fire because the patient complained of oral erosion.

**Figure 4.**
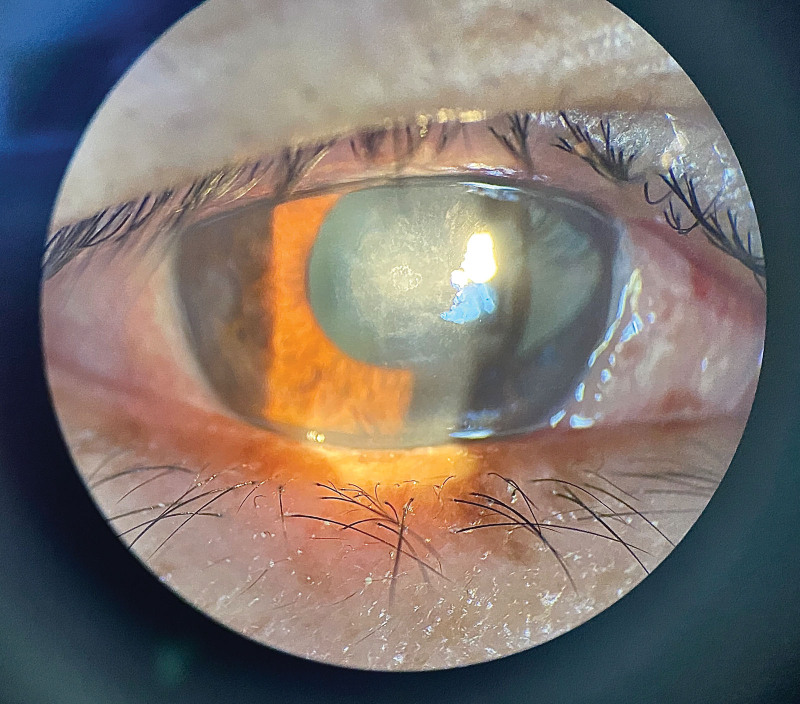
After 5 days of treatment, a slit-lamp microscope was used to check the corneal ulcer. The scope remained the same but its depth became shallow.

**Figure 5.**
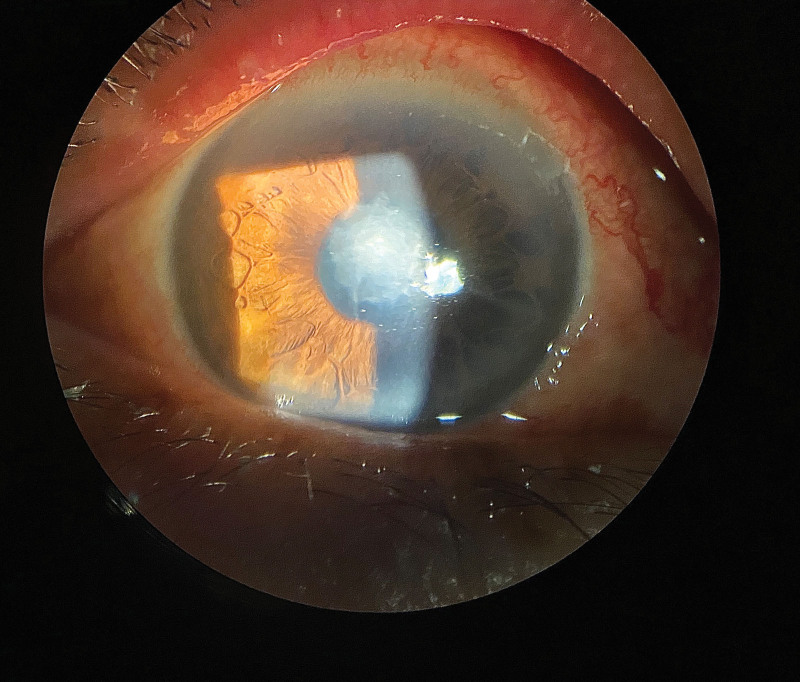
After 12 days of treatment, the scope and depth of the corneal ulcer were smaller than before.

**Figure 6.**
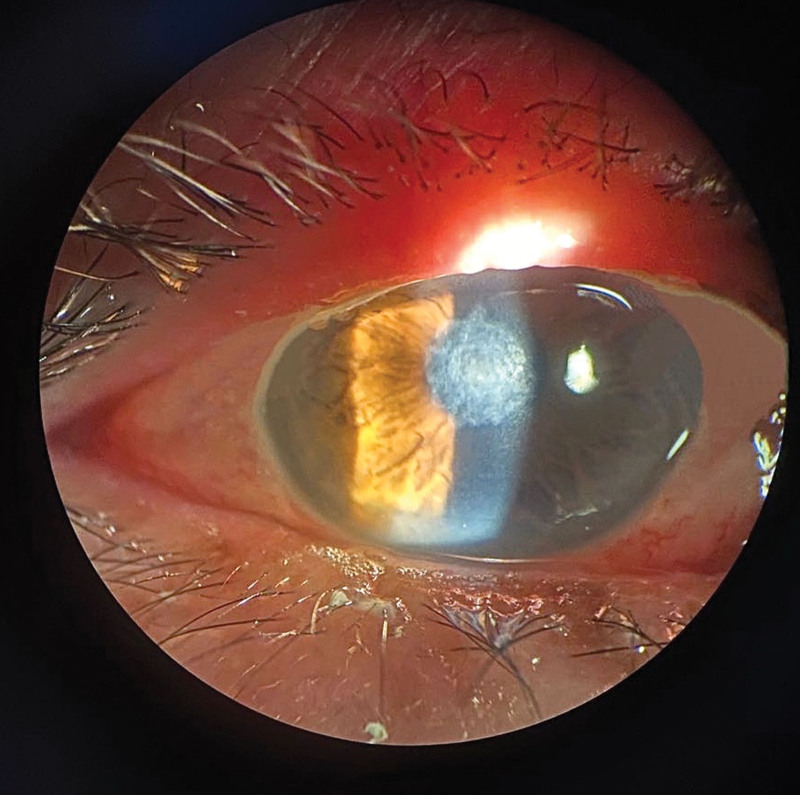
After 21 days of treatment, the corneal ulcer focus was shallower and smaller than before, as determined by slit-lamp microscopy.

**Figure 7.**
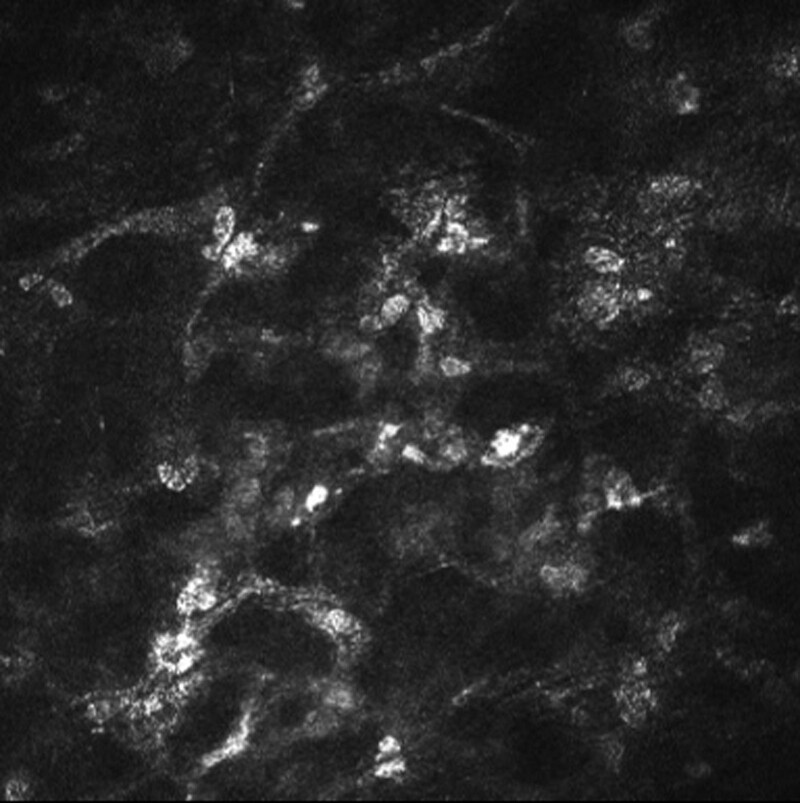
After 21 days of treatment, IVCM showed that the corneal ulcer focus had no mycelium. IVCM = in vivo confocal microscopy.

After 5 days of treatment, the patient complained that the symptoms of blurred vision did not improve significantly, and a slit-lamp microscope was used to check the corneal ulcer. The scope remained the same but its depth became shallow (Fig. 4). After 12 days of treatment, the patient reported that his vision in the affected eye had improved to 0.25. The corneal ulcer was examined using a slit-lamp microscope. The scope and depth of the corneal ulcer were smaller than before (Fig. 5). After 21 days of treatment, the vision of the affected eye was improved to 0.3, and the corneal ulcer focus was shallower and smaller than before, as determined by slit-lamp microscopy (Fig. 6). In addition, IVCM showed that the corneal ulcer focus had no mycelium (Fig. 7).

Due to the limited financial capacity, the patient and his family requested to be discharged from the hospital. Considering the stability of his condition, he was discharged with medication, and we asked the patient and his family to follow up on a regular basis.

## 3. Discussion

FK is the most damaging and visually threatening type of microbial keratitis. It is mostly caused by trauma. Fungi adhere to the corneal surface by secreting surface proteins (mainly mannoprotein). It can activate Toll-like receptors in the corneal epithelium and nucleotide-binding oligomerization domain protein receptors and release a variety of cytokines, such as interleukin-6, interleukin-8, tumor necrosis factor γ, and antibacterial peptides. These cytokines can activate corneal matrix metalloproteinase and phospholipase and damage the corneal tissue structure with mycotoxins, resulting in corneal ulcers.^[2]^ Hydrophobic proteins in fungal spores are resistant to recognition by Toll-like receptors and nucleotide-binding oligomerization domain receptors, making it difficult for the body to eliminate fungal pathogens in a timely manner.^[3]^ Therefore, strengthening the early diagnosis of FK and searching for effective antifungal treatments are essential to improving the prognosis.

In the diagnosis of FK, laboratory examination is the gold standard for the final diagnosis. However, its positive detection rate is affected by the size of the ulcer in the diseased area, the amount of material taken, and the professional level of the testers. Moreover, fungal culture requires a week or longer to determine whether there is fungal growth, which is not conducive to an early diagnosis. IVCM has obvious diagnostic advantages, especially for patients whose corneal epithelium has healed and cannot be scraped for laboratory examination.^[4]^ This case was diagnosed using IVCM combined with the patient’s history of plant trauma, symptoms, and signs.

Local antifungal drugs are currently used to treat FK, and surgery is required when the drug treatment effect is poor or the lesion involves the deep corneal stroma.^[5]^ Eye drops containing 5% natamycin or 0.1% to 0.2% amphotericin B are the treatment of choice for FK.^[6]^ Deep lamellar keratoplasty or penetrating keratoplasty can be used.^[7]^ Natamycin eye drops have obvious therapeutic effects on FK, but their relative molecular weight is high, their tissue penetration is poor, and they can cause conjunctival edema, punctate keratitis, and other adverse reactions.^[8]^ Prajna et al^[9]^ observed the effect of natamycin on filamentous keratitis and found that natamycin had poor therapeutic sensitivity in patients with moderate to severe FK, especially those with a wide range of corneal infiltration, ulcer, or near perforation. Amphotericin B is also limited in clinical use due to its toxic side effects.^[10]^ Therefore, Western medicine in the use of nonsurgical treatment of FK in a single category is ineffective. For example, before coming to our hospital, the use of natamycin eye drops resulted in the onset of pain in the patient’s eyes.

In view of the limitations of local eye drops in the treatment of FK, TCM may be beneficial. FK is called “Shiyi” TCM, and it is believed to be caused primarily by the dampness of toxins invading through injury. The syndrome types are mainly dampness-heat patterns. During the treatment, the patient’s eyes were photophobic and shed tears, and the pain was dull. The bulbar conjunctiva was slightly red, the surface of the cornea was slightly swollen, the boundary was unclear, and the color was gray. In addition, the patient experienced abdominal distension, inappetence, and diarrhea. The tongue manifestation was white fur and a pale tongue, and the pulse was slow. Therefore, the syndrome type was classified as “dampness-heat pattern.” Sanren Decoction was given to remove dampness and heat. Huangqin, Jinyinhua, and Lianqiao were added to the prescription to clear heat and purge fire because the patient had sores on the tongue. During treatment, the patient experienced mild adverse effects, high acceptance, and rapid efficacy.

We encourage clinical ophthalmologists to combine TCM and Western medicine to treat patients who are seriously ill or who cannot tolerate the side effects of local antifungal drugs. Certainly, additional observation and case accumulation are still required for the treatment of FK using a combination of TCM and Western medicine.

## Author contributions

**Data curation:** Juan Yu, Qinghua Peng.

**Funding acquisition:** Yu Huang, Juan Yu, Qinghua Peng.

**Investigation:** Yu Huang, Juan Yu, Qinghua Peng.

**Methodology:** Juan Yu, Qinghua Peng.

**Writing – original draft:** Yu Huang.

**Writing – review & editing:** Yu Huang.
